# Genetic study of the causal effect of lipid profiles on insomnia risk: a Mendelian randomization trial

**DOI:** 10.1186/s12920-023-01761-y

**Published:** 2023-12-12

**Authors:** Quancai Gong, Canshou Guo

**Affiliations:** 1 Department of Neurology, Affiliated Hospital of Jianghan University, Wuhan, 430015 Hubei China; 2 No.168, Hong Kong Road, Jiangan District, Wuhan, Hubei Province China

**Keywords:** Genetics, Insomnia, Lipid profiles, Multivariable mendelian randomization, Apolipoprotein A-1, lipoprotein-a

## Abstract

**Objectives:**

In response to the controversy surrounding observational studies of the association between lipid profiles and the risk of insomnia, the aim of this study was to analyze lipid profiles, including triglycerides (TG), apolipoprotein A-1 (ApoA-1), apolipoprotein B (ApoB) and lipoprotein A (LPA), in a European population to further assess the causal relationship between these lipid types and insomnia.

**Materials and methods:**

This study explores the causal effect of lipid profiles on insomnia based on a genome-wide association study (GWAS)-derived public dataset using two-sample and multivariate Mendelian randomization (MVMR) analysis. The main MR analyses used inverse variance weighting (IVW) odds ratio (OR), and the sensitivity analyses included weighted median (WM) and MR‒Egger.

**Results:**

Both MR and MVMR showed that lowering ApoA-1 and LPA levels had causal effects on the risk of insomnia [MR: per 10 units, ApoA-1: OR: 0.7546, 95% CI: 0.6075–0.9372, *P* = 0.011; LPA: OR: 0.8392, 95% CI: 0.7202–0.9778, *P* = 0.025; MVMR: per 10 units, ApoA-1: OR: 0.7600, 95% CI: 0.6362–0.9079, *P* = 0.002; LPA, OR: 0.903, 95% CI: 0.8283–0.9845, *P* = 0.021]. There were no causal effects of TG or ApoB on insomnia (all *P* > 0.05). The MR‒Egger intercept test, funnel plot, and IVW methods all suggested an absence of strong directional pleiotropy, and leave-one-out permutation analysis did not detect any single single-nucleotide polymorphism that had a strong influence on the results.

**Conclusion:**

Elevated levels of ApoA-1 and LPA were independently and causally associated with the risk of insomnia, suggesting that elevated ApoA-1 and LPA levels may contribute to a reduced risk of insomnia.

**Supplementary Information:**

The online version contains supplementary material available at 10.1186/s12920-023-01761-y.

## Introduction

Insomnia is a sleep disorder characterized by difficulty falling or staying asleep, and it leads to inadequate sleep that cannot meet the individual’s physiological needs [1]. This can significantly impact daytime activities and is a prevalent global disease [2, 3]. As the pace of modern life accelerates and competition has increased, psychological stress caused by social factors has also increased. This has led to an increase in the number of people suffering from insomnia [4–6]. According to epidemiological studies, the global prevalence of sleep disorders is as high as 27%, and the number of people suffering from insomnia continues to rise each year [7]. Chronic insomnia can result in fatigue, mood disorders, and other issues, creating a vicious cycle that significantly impacts the health and quality of life of those affected [8, 9].

Members of the public and medical experts are increasingly interested in understanding whether blood lipids may also play a role in causing insomnia [10–13]. Although not consistently demonstrated, lipid profiles were associated with activation of the hypothalamic‒pituitary‒adrenal axis and the sympathetic nervous system, thus providing biological evidence that lipid profiles contribute to insomnia [14–17]. Several previous studies have shown that blood lipids are associated with a significantly increased risk of insomnia [18, 19]. However, many other studies have not found a link between insomnia symptoms and these stressors [20–22]. Few studies have specifically examined the association between insomnia symptoms and dyslipidemia [23–25]. Zhang reported that compared with women without insomnia symptoms, women who reported having insomnia symptoms three or more times per week had a 25% increased risk of elevated total cholesterol [24]. In addition, previous studies have examined the association between major lipid components (including HDL cholesterol, LDL cholesterol, triglycerides and total cholesterol) and insomnia, but the causal relationship between apolipoproteins and insomnia has never been studied [26, 27]. According to relevant reports, apolipoprotein A1 (ApoA-1), apolipoprotein B (ApoB) and lipoprotein A (LPA) are closely related to the occurrence of insomnia events [28–30]. If an association between lipid profiles and insomnia symptoms does exist, it may have important implications for the management of patients with insomnia. Therefore, more extensive research is needed to fully understand this relationship.

In traditional epidemiological studies, exposure-outcome associations may be affected by unmeasured confounding and reverse causation, leading to limitations in causality inference. The Mendelian randomization (MR) method has been widely used in recent years for causality studies of genome-wide association study data [31–33]. The MR method uses the random division and combination of genetic variants during gamete formation to regroup populations randomly, which theoretically avoids the influence of confounding factors, and the variation explained by genetic variants [exposed instrumental variables (IVs)] also takes precedence over the variation explained by the outcome, thus excluding the interference of reverse causality [34–36]. To our knowledge, this is the first genetic study to investigate the causal relationship between lipid levels and insomnia in a European population using a two-sample MR method and an MVMR method with single-nucleotide polymorphisms (SNPs) as IVs and GWAS-derived data. This study aims to provide a new theoretical strategy for the diagnosis and treatment of clinical insomnia patients. Herein, we used a two-sample MR method and an MVMR method with SNPs as IVs and GWAS data to investigate the causal relationship between lipid profiles and the risk of insomnia in a European population.

## Materials and methods

### Selection of data sources

In this study, lipid profiles (TG, ApoA-1, ApoB, LPA) and insomnia were analyzed by two-sample MR and MVMR using pooled statistics from a large GWAS. All raw GWAS were approved by a responsible ethics committee, and participants signed informed consent [37]. However, the present study did not require ethical approval because it was derived from summary statistics.

Genetic datasets of lipid profiles (TG, ApoA-1, ApoB, LPA) were obtained from the largest UK Biobank (UKB) GWAS [sample size: TG (ID: ieu-b-4850): N = 78,700; ApoA-1 (ID: met-c-842): N = 20,687; ApoB (ID: met-c-843): N = 20,690; LPA (ID: ukb-d-30790_irnt): N = 439,214] and included European Caucasian participants. Supplementary Table 1 provides comprehensive information on the data sources for the exposure and outcome samples. The linkage disequilibrium (LD) among selected SNPs was tested within the condition of r^2^ = 0.001, clumping window = 10,000 kb, pop="1000G EUR” to minimize the impact of strong LD. As primary IVs for the MR analysis, 54, 14, 28, and 63 SNPs associated with TG, ApoA-1, ApoB, and LPA, respectively, with genome-wide significance (*P* < 5 × 10^− 8^) in the studies of the GWAS Catalog were utilized. To eliminate the confounding effect of obesity, the main IVs were adjusted by removing all the obesity-related SNPs. This was done because several studies have demonstrated that obesity is a major contributor to insomnia, and its occurrence is closely linked to lipid metabolism [22, 38–41]. In this study, PhenoScanner (www.phenoscanner.medschl.cam.ac.uk), a human genotype-phenotype association database, was used to search for traits of preselected SNPs. Specifically, several potential confounding factors were associated with obesity, including body mass index, total body fat mass, body weight, and body fat percentage. The IVs TG, ApoA-1, ApoB, and LPA were screened for SNPs using PhenoScanner. SNPs that were not found by PhenoScanner and those associated with potential confounders were removed. As a result, 41, 12, 18, and 43 SNPs remained for TG, ApoA-1, ApoB, and LPA, respectively. For more information on the screened SNPs associated with potential confounding factors, please refer to Supplementary Table 2.

**Fig. 1:**
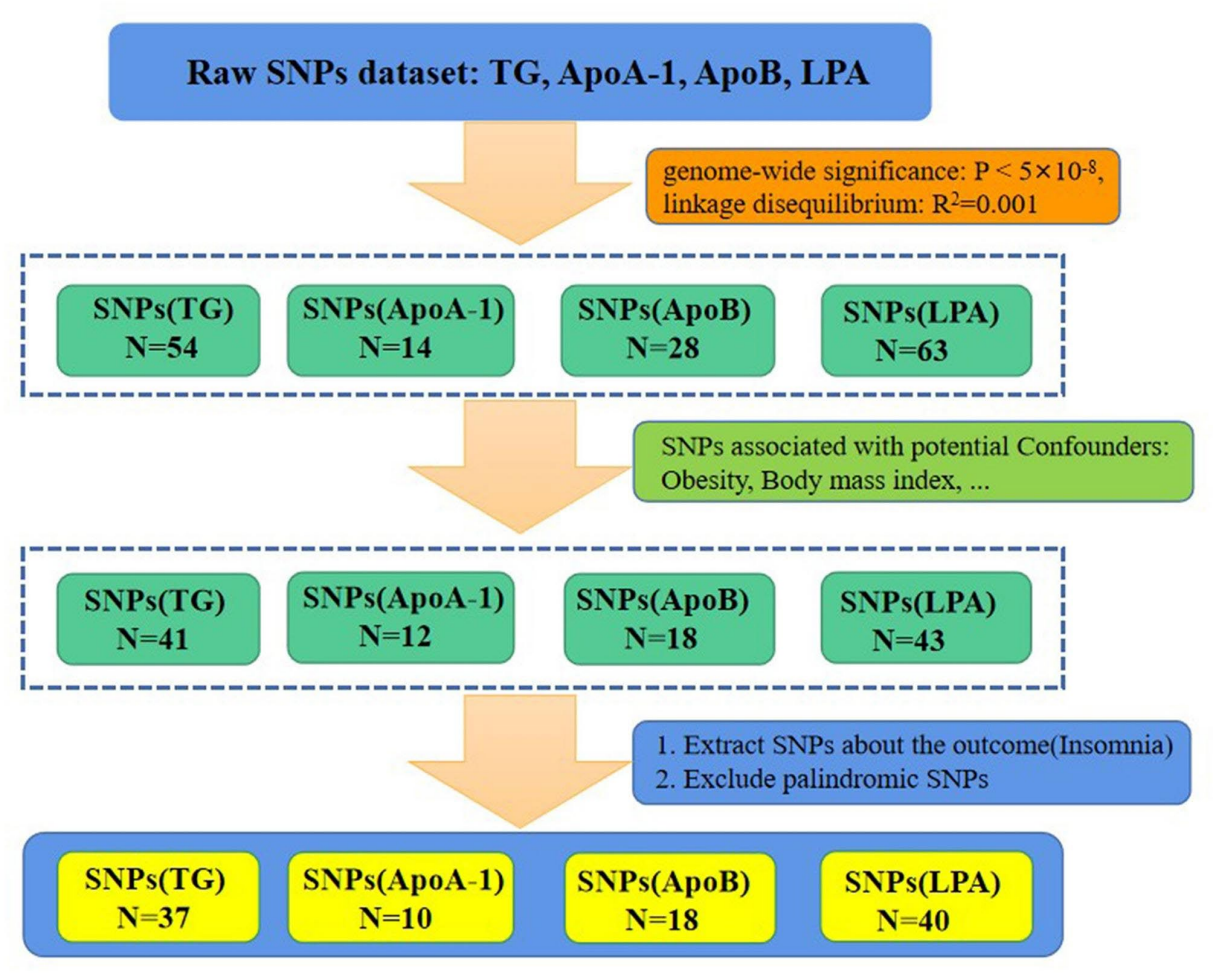
The overall process for single-nucleotide polymorphism (SNP) extraction

We used the keyword “insomnia” in the MR-data database and chose data from participants with European ancestry. The insomnia dataset with the largest sample size (dataset ID: finn-b-F5_INSOMNIA, N = 153,006, diagnosis: insomnia) was specifically selected. In the insomnia dataset, we extracted the SNPs of the IVs for the lipid profile, which included TG, ApoA-1, ApoB, and LPA. After excluding the palindromic SNPs, 37, 10, 18, and 40 SNPs were obtained for each respective variable. The entire process of SNP extraction is illustrated in Fig. 1, and the final extracted SNPs can be found in Supplementary Table 3.

### Mendelian randomization analysis

**Fig. 2:**
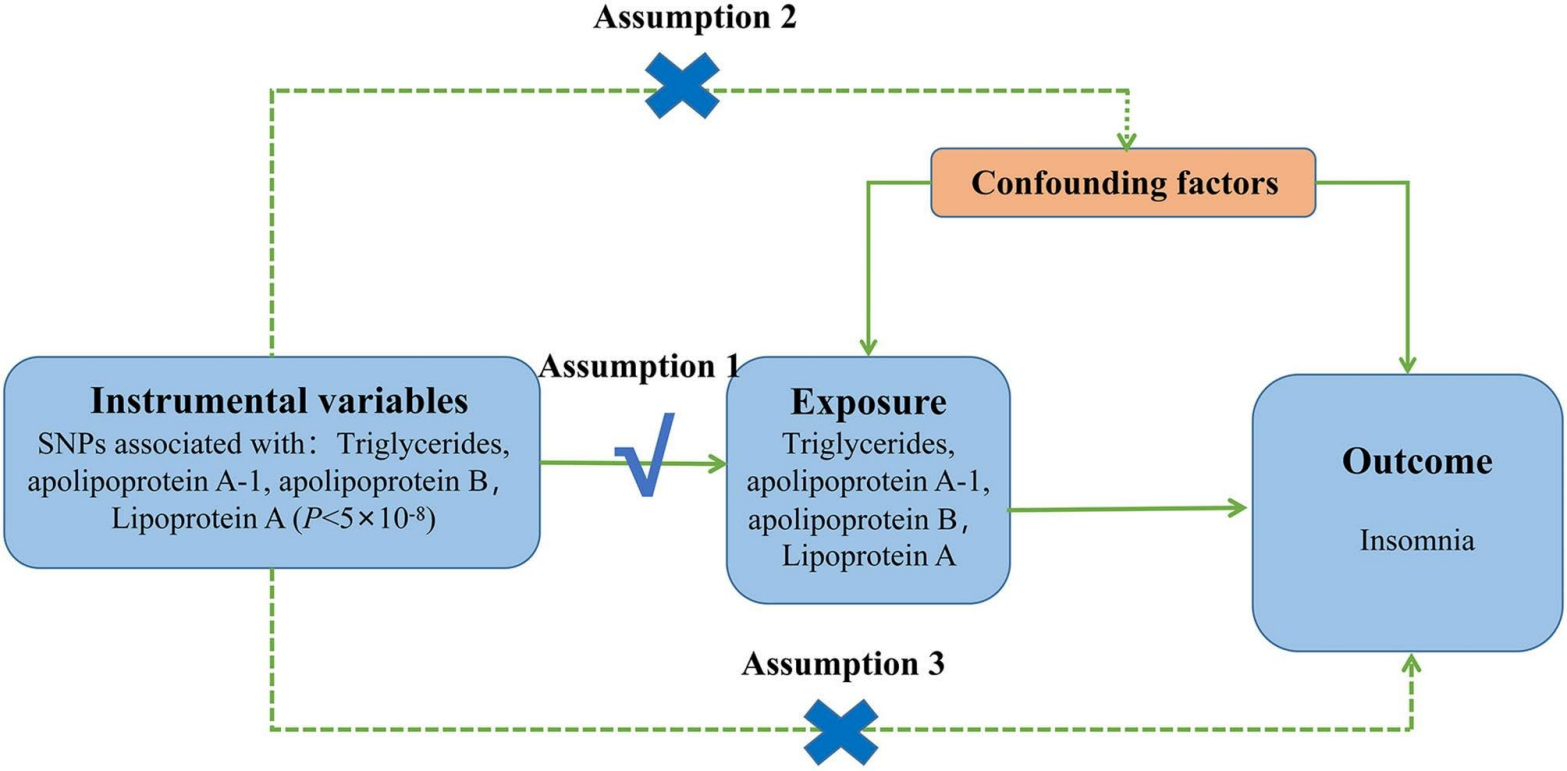
Schematic representation of two-sample Mendelian randomization

To investigate a possible causal relationship between blood lipids (TG, ApoA-1, ApoB, LPA) and the risk of insomnia, we performed five independent MR analyses on two samples. These analyses are based on three main assumptions [42, 43]: (1) there is a strong association between genetic variables and exposure; (2) variables affect outcomes only through their effect on exposure; and (3) variables are conditionally independent of the outcome given the exposure and the confounding factors. Please refer to Fig. 2 for further details.

Three MR methods, IVW, MR-egger and WM, were used using version R 4.2.2 (www.r-project.org) of the “TwoSampleMR” package (https://github.com/MRCIEU/TwoSampleMR). The results are expressed as the mean effect (log-transformed) and 95% confidence interval (95% CI) of lipid profile increase per 1 SD genetic prediction. The visualization results are shown in the scatter plot. In addition, *P* < 0.05 was considered to indicate statistical significance. The results were visually presented through leave-one-out, scatter, forest and funnel plots.

**Fig. 3:**
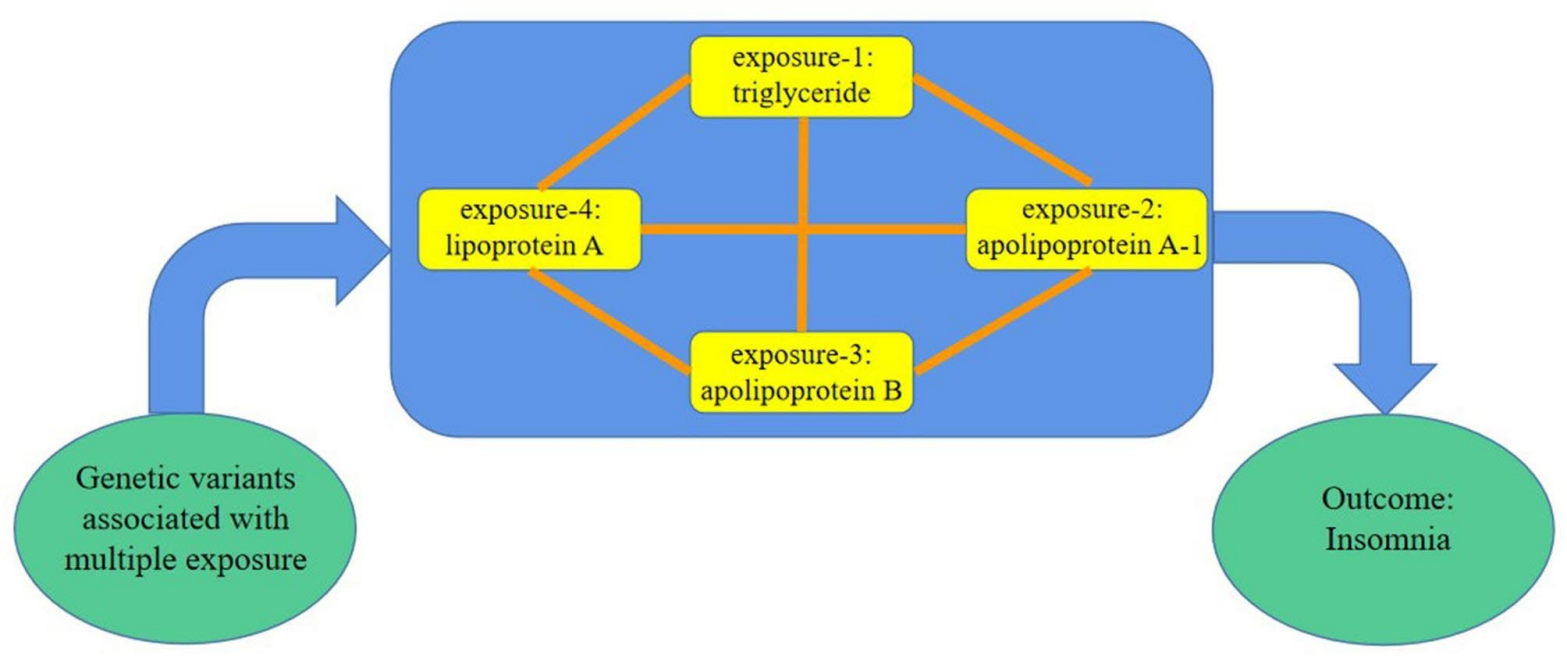
Schematic representation of multivariable Mendelian randomization

To eliminate confounding factors, multivariate Mendelian randomization (MVMR) analyses were conducted on selected exposures, including TG, ApoA-1, ApoB, and LPA. All SNPs with LD (R^2^ = 0.001, clumping window = 10,000) and SNPd with intermediate allele frequencies less than 0.01 were excluded, leaving a total of 65 SNPs. The interactions among these exposures were also eliminated during the analysis (Fig. 3).

### Sensitivity analysis

Cochran’s Q test was derived from the IVW estimates and used to explore potential heterogeneity between IVs. Funnel plots were used to assess whether there was direct heterogeneity between IVs. When significant heterogeneity (*P* < 0.05) was detected, the IVW model results were used to detect it. The MR‒Egger regression-derived MR‒Egger intercept was used to examine directional pleiotropy of IVs, with *P* < 0.05 as the threshold for significant pleiotropy. The following three principles should be followed in the selection of the MR method [42, 43]: (1) on the premise of no heterogeneity or pleiotropy, the estimated results of the IVW method should be preferentially used; (2) when only heterogeneity is used, the results of the WM method are preferred, and the random effects model of IVW can also be used; and (3) if there is pleiotropy, the MR‒Egger method is preferred for calculating the results. The robustness of the IVs was tested using a leave-one-out approach and shown in leave-one-out plots.

## Results

### Mendelian randomization estimates

**Fig. 4:**
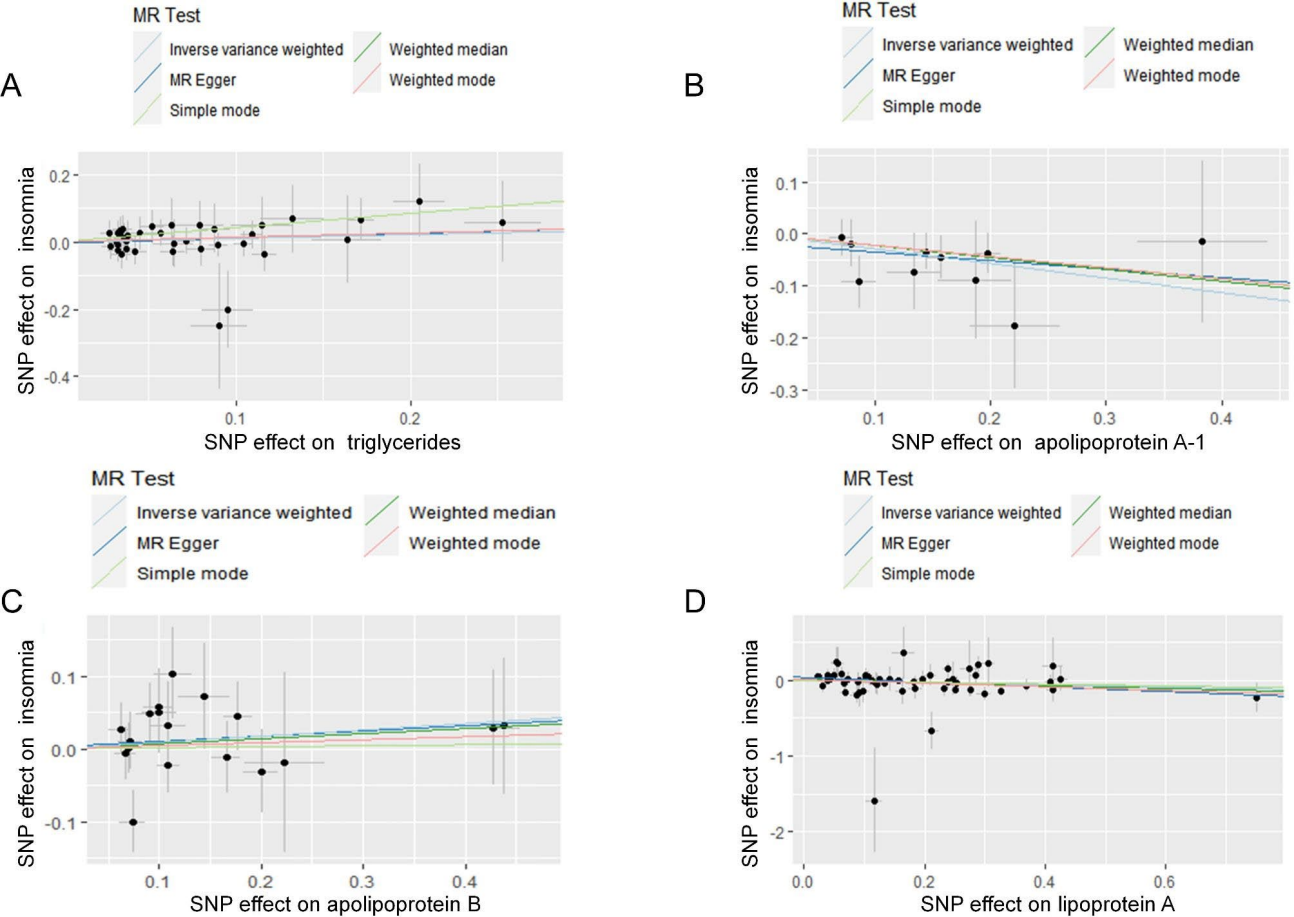
Scatter plot. The effect of triglycerides **(A)**, apolipoprotein A-1 **(B)**, apolipoprotein B **(C)** and lipoprotein-a **(D)** on insomnia risk. The slope represents the magnitude of the causal effect

**Fig. 5:**
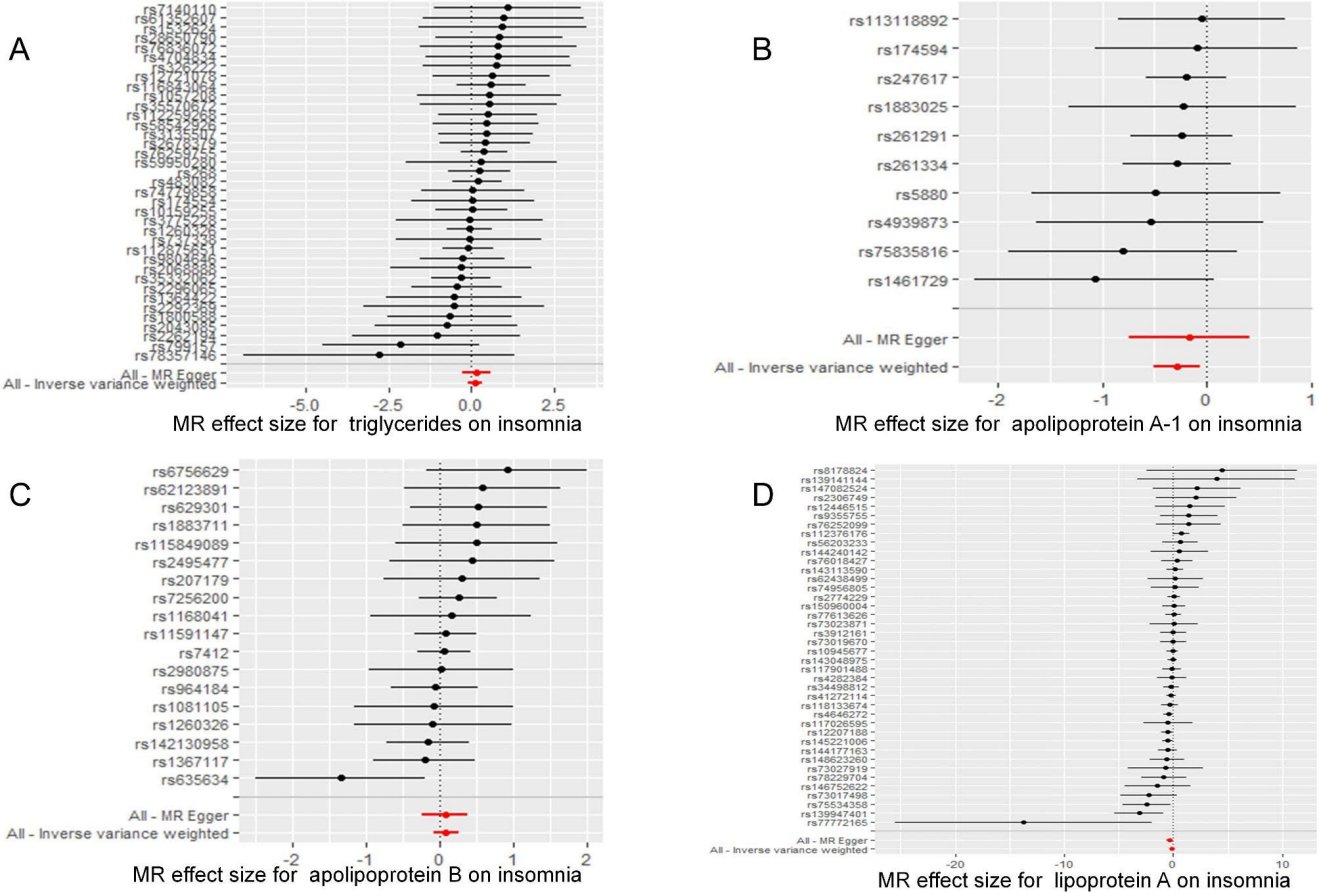
Forest plot to assess the causal effect of individual SNP on insomnia risk. **(A)** Triglyceride, **(B)** apolipoprotein A-1, **(C)** apolipoprotein B, **(D)** lipoprotein-a

**Fig. 6:**
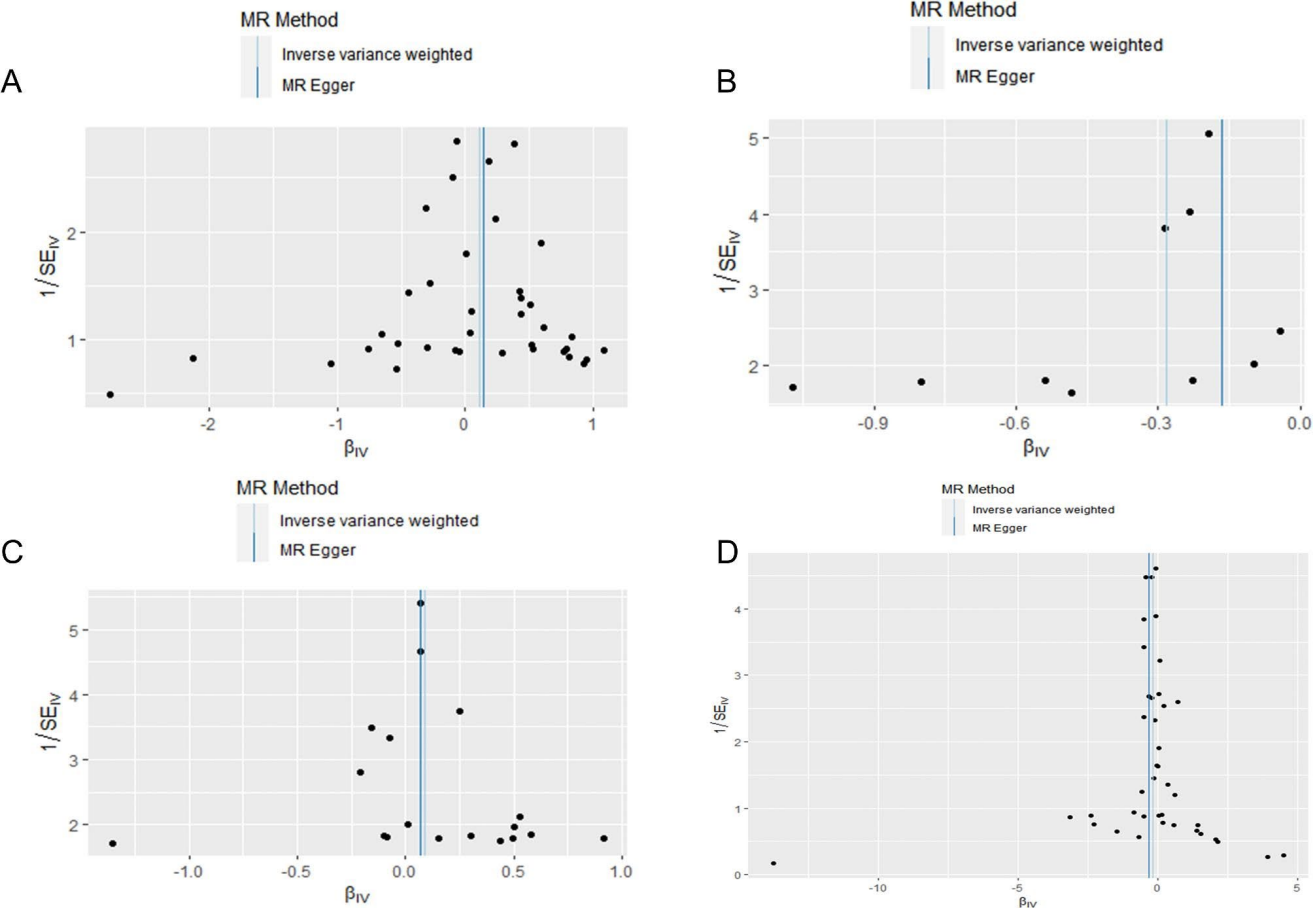
Funnel plot to assess the heterogeneity of the effects of triglycerides **(A)**, apolipoprotein A-1 **(B)**, apolipoprotein B **(C)** and lipoprotein-a **(D)** on insomnia risk

**Fig. 7:**
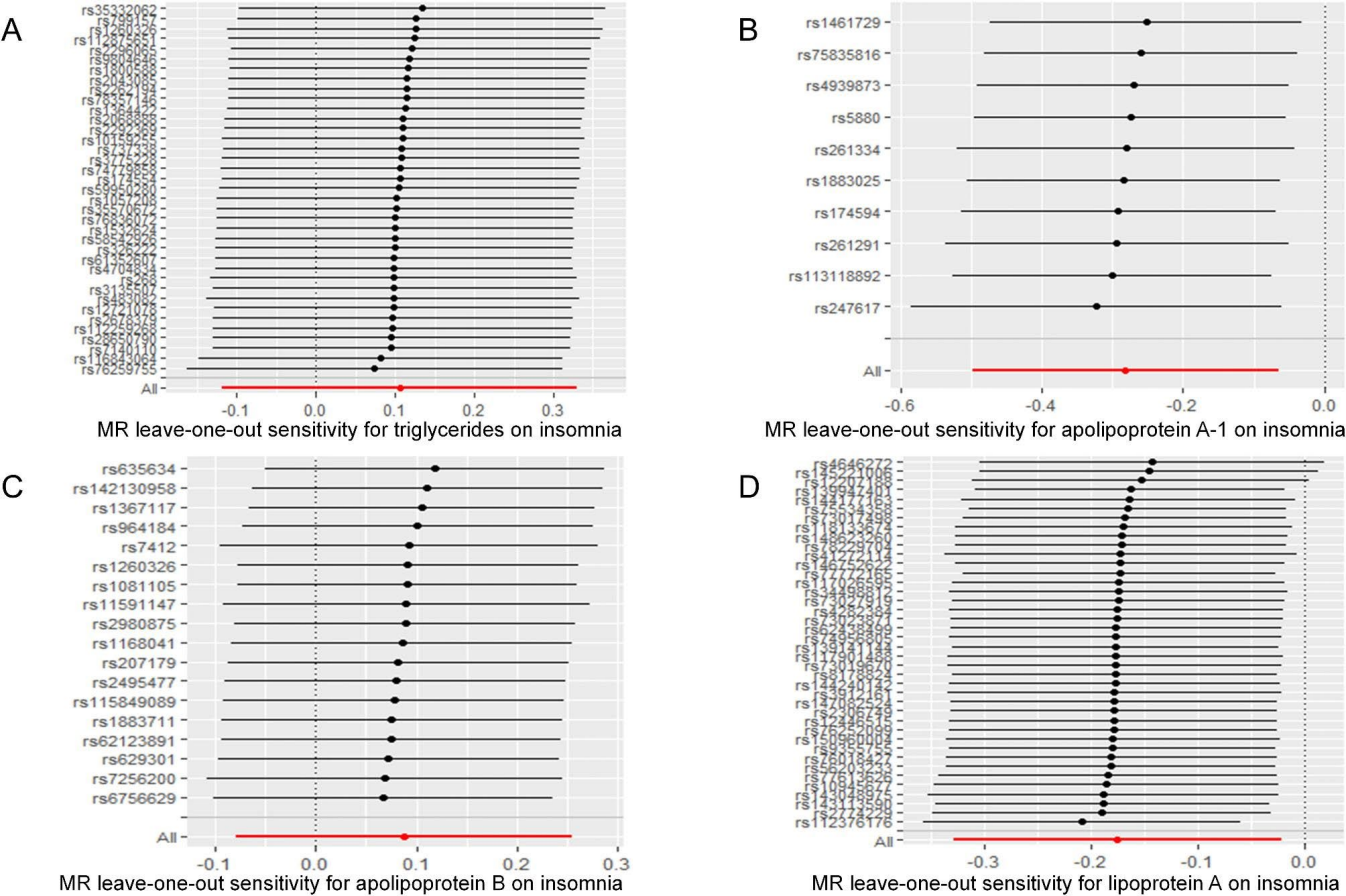
Leave-one-out analysis of the effect of triglycerides **(A)**, apolipoprotein A-1 **(B)**, apolipoprotein B **(C)** and lipoprotein-a **(D)** on insomnia risk

Table 1 displays the estimated correlation between the lipid profile and the likelihood of experiencing insomnia. The results of the two-sample MR analysis demonstrated that the levels of ApoA-1 were causally linked to the risk of insomnia, as determined by IVW. The odds ratio (OR) indicated that a one-SD increase in ApoA-1 was causally related to a 24.54% decrease in the risk of insomnia (Table 1, ApoA-1: N = 10 SNPs, OR: 0.7546, 95% CI: 0.6075–0.9372, *P* = 0.011). According to IVW analysis, there is a causal relationship between the levels of LPA and the risk of insomnia. Additionally, the results showed that a one-SD increase in LPA can lead to a 16.08% reduction in the risk of insomnia (Table 1, LPA: N = 40 SNPs, OR: 0.8392, 95% CI: 0.7202–0.9778, *P* = 0.025). The current study did not find any significant correlation between other lipids, such as TG and ApoB, and the risk of insomnia (Table 1, TG: N = 37 SNPs, OR: 1.1114, 95% CI: 0.8880–1.3910, *P* = 0.356; ApoB: N = 18 SNPs, OR: 1.0911, 95% CI: 0.9231–1.2896, *P* = 0.307). According to the MVMR analysis, there is a causal relationship between an increase in ApoA-1 and a decreased risk of insomnia. The OR of MVMR suggests that a one-SD increase in ApoA-1 is associated with a 24.00% reduction in the risk of insomnia (Table 2, ApoA-1, OR: 0.7600, 95% CI: 0.6362–0.9079, *P* = 0.002). Additionally, the causal effect of LPA on insomnia was still significant in the MVMR analysis (LPA, OR: 0.903, 95% CI: 0.8283–0.9845, *P* = 0.021). The study utilized scatterplots, forest plots, and funnel plots to analyze the causal relationship between ApoA-1, LPA, and the risk of insomnia. The scatterplot in Fig. 4 demonstrates this relationship. Forest plots in Figs. 5 and 6 show the causal effects of ApoA-1 and LPA on insomnia risk, respectively, and all funnel plots displayed no asymmetry. The leave-one-out plots in Fig. 7 indicated that even with the removal of one SNP from the IVs, the remaining data still yielded a significant causal effect. Additionally, all IVs had *F*-statistics greater than 30.

### Sensitivity analysis

There was no heterogeneity observed in the Q variable for TG, ApoA-1, ApoB, LPA, and insomnia (Table 3, MR‒Egger Q, TG: *P* = 0.996, ApoA-1: *P* = 0.892, ApoB: *P* = 0.601, LPA: *P* = 0.339). Additionally, MR‒Egger intercepts did not indicate any significant horizontal pleiotropy in any of the aforementioned analyses (Table 3, MR‒Egger intercept, TG: *P* = 0.850, ApoA-1: *P* = 0.676, ApoB: *P* = 0.906, LPA: *P* = 0.122).

## Discussion

In this study, two-sample MR and MVMR methods were used to investigate the association between serum lipid levels (including TG, ApoA-1, ApoB, LPA) and insomnia in the European population. The results revealed a causal relationship between ApoA-1 and LPA levels and insomnia in both two-sample MR and MVMR.

Previous studies have shown that insomnia affects the metabolic rate and lipid profile stability. Sleep deprivation also affects hormone regulation, including increased cortisol and gastrin and decreased leptin and glucose metabolism [44, 45]. Although several studies have investigated risk factors for dyslipidemia, such as diet, obesity, and lifestyle [46–48], few studies have extensively investigated the relationship between insomnia and abnormal apolipoprotein levels (including ApoA-1, ApoB and LPA). Several recent studies have investigated the relationship between insomnia and blood lipids, but the results have been inconsistent [31, 49].

Insomnia is a common sleep disorder. Symptoms include trouble falling asleep, difficulty staying asleep, waking up early, and poor sleep quality [1, 2]. Although the physiological and psychosocial effects of insomnia have been extensively studied [50], the relationship between insomnia and lipid profiles is unclear.

As mentioned above, the validity of causal estimates is most effective when the three assumptions of the MR model are met. First, 14 and 63 significantly correlated and independent SNPs were selected that were closely related to ApoA-1 and LPA, respectively. Additionally, the selected SNPs with F values greater than 30 are considered strong IVs [51, 52]. In addition, the SNP data in this study were all from European populations, which to some extent avoided the bias caused by different populations. To better address the bias caused by pleiotropy in studies of MR, SNPs associated with other confounders were identified by using PhenoScanner. Obesity, body mass index and other indicators are to some extent risk factors for insomnia [53]. Therefore, removing these SNPs, which are strongly associated with confounding factors, does not change the results of the MR estimates. In addition, in this study, the intercept of the MR–Egger method was close to 0 (*P* > 0.05), indicating that there was no pleiotropy due to unknown factors. Finally, in the leave-one-out sensitivity analysis, no individual SNP had a significant impact on the overall efficacy of the assessment. Two-sample MR is used to evaluate the causal effect of a single exposure on a single outcome variable. It is one of the simplest and most common methods of MR analysis. MVMR considers the causal effects of multiple exposure factors on one or more outcome variables. It allows multiple causal paths to be evaluated simultaneously and addresses confounding issues between multiple causal factors. In this study, since the levels of the four lipids we selected may interact with each other, we selected MVMR for analysis to increase the credibility of the conclusion. By MVMR, the results revealed a significant causal relationship between genetic gain in ApoA-1 and LPA and risk of insomnia, but not between TG and ApoB. This result supports the determination of causality. Therefore, the selected SNPs and the study results are reliable.

LPA is a specific plasma lipoprotein composed of apolipoprotein (a) and a low-density lipoprotein-like lipoprotein particle. The serum concentration was mainly determined by the number of Kringle IV nucleotide repeats in the LPA gene [54, 55]. The more Kringle IV copies there are, the larger the LPA molecule synthesized by the body and the lower the serum level [56, 57]. Conversely, the smaller the LPA molecule is, the higher the serum level [58]. LPA levels are therefore relatively stable in individuals and are basically unaffected by age, sex, weight, environment and most existing cholesterol-lowering drugs [59]. However, LPA levels vary greatly between individuals, up to a factor of 1,000, and there are significant racial differences [60]. The specific physiological function of LPA has not been fully defined, but existing research has shown that LPA has complex and diverse properties. Among behavioral risk factors, serum lipid levels, especially LPA, are considered important in influencing sleep quality, duration and disturbances. However, evidence on the relationship between insomnia and dyslipidemia is sparse and conflicting, and information on LPA and apolipoproteins is lacking [61–63]. In a small sample of Taiwanese communities, Professor Chien found that people who experienced insomnia symptoms almost every day had significantly lower total cholesterol levels than those who rarely experienced insomnia symptoms. No clear trend between insomnia severity and LDL-c (low-density lipoprotein cholesterol), TG and HDL-c (high-density lipoprotein cholesterol) levels was observed [23].

In this study, a significant causal relationship between serum LPA levels and insomnia in the European population was found, with each SD increase in LPA levels reducing the risk of insomnia by 16.08%, suggesting that serum LPA levels are a protective factor against insomnia. This is in line with Chien’s findings [23]. ApoA-1 is the major protein skeleton of HDL, accounting for approximately 70% of HDL structural proteins [64, 65]. Its main function is to be responsible for the assembly of HDL and to return excess cholesterol from peripheral tissues to the liver through the process of cholesterol reverse transport, thus playing an important role in regulating cholesterol homeostasis in the body [66, 67]. In this study, a significant causal relationship between serum ApoA-1 levels and insomnia in the European population was found, with each SD increase in ApoA-1 levels reducing the risk of insomnia by 24.54%, suggesting that serum ApoA-1 levels are also a protective factor for insomnia.

Previous studies have confirmed that serum triglyceride levels do not correlate significantly with insomnia, which is consistent with the results of this study [66, 67]. The potential reasons why ApoA-1 and LPA affect sleep quality while TG does not require further analysis. Their different structures may explain part of the reason: ApoA-1 is the major apolipoprotein of HDL, and its main function is to transport cholesterol, not TG. Fluctuations in ApoA-1 levels have a direct effect on cholesterol levels but not on TG [68]. The total lipid content of HDL includes 12% TG and 40% cholesterol lipids [69]. Cholesterol is the main core lipid of HDL and plays a crucial role in maintaining proper membrane permeability and fluidity [70]. Cholesterol is also essential for the production of steroid hormones, bile acids, and vitamin D. Unlike triglycerides, cholesterol has hydrophilic groups that do not easily accumulate [71]. Therefore, the different physiological structures may be one of the reasons why apolipoproteins and TG have different biological roles, and these underlying mechanisms and potential common genetic factors require further elucidation in future prospective studies.

### Strengths of the study

In this study, for the first time, the causal relationship between lipid levels and insomnia at the genetic level in a European population was analyzed using a two-sample MR method and an MVMR method with SNPs as IVs and GWAS statistics. As previously mentioned, MR analyses have the advantage of excluding the unreliability of results due to confounding factors and reverse causality between exposure and outcome that occur in traditional observational studies.

### Limitations of the study

There are several limitations to this study. The MR analysis performed here requires a clear understanding of gene-disease and gene-intermediate phenotype relationships to minimize confounding bias due to horizontal or vertical gene pleiotropy. However, due to the complex functions of these genes and their potential to influence a range of metabolic pathways and other biological activities through poorly understood mechanisms, fully controlling for such confounding effects is challenging. In addition, although this study included a large overall sample size, there are limitations in the publicly available GWAS databases used, such that stratification based on age or other variables was not possible. This limitation inhibited efforts to estimate the association between lipid profiles and insomnia. The databases used for this study were specific to a European population, and whether these results can be generalized to populations of other ethnicities will also require further clinical validation. Finally, although the effects of confounding factors such as race and obesity were excluded, there was a lack of MR analysis for subgroups such as sex and age to assess the causal relationship between lipid profiles and insomnia.

## Conclusions

This study employed two-sample MR and MVMR analyses to demonstrate the independent causal effect of genetic reduction of ApoA-1 and LPA on the risk of insomnia while avoiding the confounding effect of obesity. This study suggests that higher levels of ApoA-1 and LPA may contribute to a reduced risk of insomnia. However, contrary to conventional knowledge, there does not appear to be an independent causal relationship between serum TG and ApoB levels and the risk of insomnia. Therefore, strategies to improve insomnia by controlling triglyceride levels require further research. In conclusion, this study proposes that controlling serum ApoA-1 and LPA may help to improve the incidence of insomnia and thus provides a theoretical basis for the clinical treatment of patients with insomnia. These results are limited to predictions at the genetic level only; therefore, additional clinical studies are needed for confirmation.

**Table 1:** Mendelian randomization estimates lipid profiles and insomnia

Traits	Methods	Nsnp	OR(95%CI)	*P*-Value
**TG**	MR-Egger regression	37	1.1505(0.7552, 1.7528)	0.518
	Weighted median		1.1165(0.8173, 1.5252)	0.489
	IVW(fixed effect)		1.1114(0.8880, 1.3910)	0.356
**ApoA-1**	MR-Egger regression	10	0.8484(0.4784, 1.5035)	0.589
	Weighted median		0.7945(0.6063, 1.0410)	0.095
	IVW(fixed effect)		0.7546(0.6075, 0.9372)	0.011
**ApoB**	MR-Egger regression	18	1.0743(0.7930, 1.4554)	0.650
	Weighted median		1.0725(0.8542, 1.3465)	0.547
	IVW(fixed effect)		1.0911(0.9231, 1.2896)	0.307
**LPA**	MR-Egger regression	40	0.7183(0.5626, 0.9170)	0.012
	Weighted median		0.8605(0.7033, 1.0528)	0.144
	IVW(fixed effect)		0.8392(0.7202, 0.9778)	0.025

**Note**: IVW, inversevariance weighted; OR: odds ratio; 95%CI, 95% confidence interval; *P* < 0.05 is considered statistically significant

**Table 2:** Multivariable Mendelian randomization estimates lipid profiles and insomnia

Traits	N = 65	OR	95% CI	*P*-Value
**TG**		0.8892	(0.6808, 1.1614)	0.389
**ApoA-1**		0.7600	(0.6362, 0.9079)	0.002
**ApoB**		1.3051	(0.9914, 1.7181)	0.058
**LPA**		0.9030	(0.8283, 0.9845)	0.021

**Note**: OR: odds ratio; 95%CI, 95% confidence interval; *P* < 0.05 is considered statistically significant

**Table 3:** The estimates of Q test and MR-Egger intercept

Traits	Q test (Q *P*-Value)	MR-Egger intercept (*P*-Value)
**TG**	0.996	(−0.003)0.850
**ApoA-1**	0.892	(−0.018)0.676
**ApoB**	0.601	(0.002)0.906
**LPA**	0.339	(0.033)0.122

Note: Cochran’s Q test was derived from the IVW estimates and used to explore potential heterogeneity between IVs; The MR‒Egger regression-derived MR‒Egger intercept was used to examine directional pleiotropy of IVs, with *P* < 0.05 as the threshold for significant pleiotropy

### Electronic supplementary material

Below is the link to the electronic supplementary material.

Supplementary Material 1

Supplementary Material 2

Supplementary Material 3

## Data Availability

The data and material that support the findings of this study are available from public datasets that can be found in the GWAS Catalog (https://www.ebi.ac.uk/gwas/) and IEU OpenGWAS (https://gwas.mrcieu.ac.uk/datasets/).

## Abbreviations

ApoA-1: apolipoprotein A-1
ApoB: apolipoprotein B
CI: confidence interval
GWAS: genome-wide association studies
HDL-c: high-density lipoprotein cholesterol
IVs: instrumental variables
IVW: inverse variance weighting
LD: linkage disequilibrium
LDL-c: low-density lipoprotein cholesterol
LPA: lipoprotein A
MR: Mendelian randomization
MVMR: multivariate Mendelian randomization
OR: odds ratio
SNPs: single nucleotide polymorphisms
TG: triglycerides
UKB: UK Biobank
WM: weighted median
