# Supplementary material for: Genetic study of the causal effect of lipid profiles on insomnia risk: a Mendelian randomization trial
Source: BMC Med Genomics. 2023 Dec 12;16:325. doi: 10.1186/s12920-023-01761-y (PMC10714578; doi:10.1186/s12920-023-01761-y)
Supplement: Supplementary file 1 — Supplementary Material 1 [file 12920_2023_1761_MOESM1_ESM.docx]

|  |  | **Sample size** | **Year** | **SNP number** | **PMID** | **Authors** | **ID** |
| --- | --- | --- | --- | --- | --- | --- | --- |
| **Exposure** | Ttriglycerides | 78,700 | 2022 | 7,892,037 | 35534559 | Howe LJ | ieu-b-4850 |
|  | apolipoprotein A-1 | 20,687 | 2016 | 11,760,646 | 27005778 | Kettunen | met-c-842 |
|  | apolipoprotein B | 20,690 | 2016 | 11,813,266 | 27005778 | Kettunen | met-c-843 |
|  | lipoprotein A | 439,214 | 2018 | 13,583,854 | NA | Neale lab | ukb-d-30790_irnt |
| **Outcome** | Insomnia | 217,855 | 2021 | 16,380,465 | NA | FinnGen | finn-b-F5_INSOMNIA |

**Supplementary Table1. Information of the data sources for exposure and outcomes included in this Mendelian randomization study.**
