# Supplementary material for: Genetic study of the causal effect of lipid profiles on insomnia risk: a Mendelian randomization trial
Source: BMC Med Genomics. 2023 Dec 12;16:325. doi: 10.1186/s12920-023-01761-y (PMC10714578; doi:10.1186/s12920-023-01761-y)
Supplement: Supplementary file 2 — Supplementary Material 2 [file 12920_2023_1761_MOESM2_ESM.docx]

| **Supplementary Table 2. Removal of single nucleotide polymorphisms (SNPs) associated with potential confounders.** | | | | | | | | |
| --- | --- | --- | --- | --- | --- | --- | --- | --- |
|  |  |  | **Potential confounders** | | | | |  |
| **chr** | **SNP** | **Traits** | **Obesity** | **body mass index** | **Whole body fat mass** | **Body fat percentage** | **Weight** | **No searching results** |
| 1 | rs35169323 | Triglyceride |  |  |  |  |  | √ |
| 1 | rs2943660 | Triglyceride |  |  |  | √ |  |  |
| 1 | rs1128249 | Triglyceride |  |  |  | √ |  |  |
| 1 | rs40270 | Triglyceride |  |  |  | √ |  |  |
| 1 | rs9272729 | Triglyceride |  |  |  |  |  | √ |
| 1 | rs987763 | Triglyceride |  |  | √ |  |  |  |
| 2 | rs4273864 | Triglyceride |  |  |  |  |  | √ |
| 2 | rs11231693 | Triglyceride |  | √ |  |  |  |  |
| 2 | rs71480306 | Triglyceride |  |  |  |  | √ |  |
| 2 | rs10773049 | Triglyceride |  |  | √ | √ |  |  |
| 2 | rs147233090 | Triglyceride |  |  | √ | √ |  |  |
| 3 | rs3093680 | Triglyceride |  |  |  |  |  | √ |
| 3 | rs6070491 | Triglyceride |  |  |  |  |  | √ |
| 4 | rs144064722 | ApoA-1 |  |  |  |  |  | √ |
| 15 | rs6494025 | ApoA-1 |  |  | √ |  |  |  |
| 1 | rs3005923 | ApoB |  |  |  |  |  | √ |
| 1 | rs143341434 | ApoB |  |  | √ | √ |  |  |
| 1 | rs17395160 | ApoB |  |  |  |  |  | √ |
| 1 | rs190934192 | ApoB |  |  |  |  |  | √ |
| 4 | rs144064722 | ApoB |  |  | √ |  |  |  |
| 4 | rs182695896 | ApoB |  |  |  |  |  | √ |
| 5 | rs10056811 | ApoB |  | √ |  |  |  |  |
| 7 | rs4722043 | ApoB |  |  |  |  |  | √ |
| 19 | rs150617279 | ApoB |  |  |  |  |  | √ |
| 19 | rs137992968 | ApoB |  |  |  |  |  | √ |
| 6 | rs73592974 | Lipoprotein A |  |  |  |  |  | √ |
| 6 | rs1317026 | Lipoprotein A |  |  |  |  |  | √ |
| 6 | rs6902571 | Lipoprotein A |  |  |  |  |  | √ |
| 6 | rs77925194 | Lipoprotein A |  |  |  |  |  | √ |
| 6 | rs12210290 | Lipoprotein A |  |  |  |  |  | √ |
| 6 | rs150489934 | Lipoprotein A |  |  |  |  |  | √ |
| 6 | rs79847831 | Lipoprotein A |  |  |  |  |  | √ |
| 6 | rs112820829 | Lipoprotein A |  |  |  |  |  | √ |
| 6 | rs76037141 | Lipoprotein A |  |  |  |  |  | √ |
| 6 | rs76763527 | Lipoprotein A |  |  |  |  |  | √ |
| 6 | rs147626015 | Lipoprotein A |  |  |  |  |  | √ |
| 6 | rs71567603 | Lipoprotein A |  |  |  |  |  | √ |
| 6 | rs9355839 | Lipoprotein A |  |  |  |  |  | √ |
| 6 | rs146184004 | Lipoprotein A |  |  |  |  |  | √ |
| 6 | rs559843888 | Lipoprotein A |  |  |  |  |  | √ |
| 6 | rs117612826 | Lipoprotein A |  |  |  |  |  | √ |
| 6 | rs9365059 | Lipoprotein A |  |  |  |  |  | √ |
| 6 | rs76993980 | Lipoprotein A |  |  |  |  |  | √ |
| 6 | rs1247306 | Lipoprotein A |  |  |  |  |  | √ |
| 6 | rs376402211 | Lipoprotein A |  |  |  |  |  | √ |
