# Supplementary material for: Genetic study of the causal effect of lipid profiles on insomnia risk: a Mendelian randomization trial
Source: BMC Med Genomics. 2023 Dec 12;16:325. doi: 10.1186/s12920-023-01761-y (PMC10714578; doi:10.1186/s12920-023-01761-y)
Supplement: Supplementary file 3 — Supplementary Material 3 [file 12920_2023_1761_MOESM3_ESM.docx]

| **Supplementary Table 3. Final extracted SNPs.** | | | | | | | | | | | | |
| --- | --- | --- | --- | --- | --- | --- | --- | --- | --- | --- | --- | --- |
|  |  |  |  |  | **Lipid** | | | | **Insomnia** | | | |
| **SNP** | **exposure** | **Mapped gene** | **effect_allele** | **other_allele** | **beta.exposure** | **se.exposure** | **pval.exposure** | **eaf.exposure** | **beta.outcome** | **se.outcome** | **eaf.outcome** | **pval.outcome** |
| rs10159255 | Triglyceride | DOCK7 | A | C | -0.0713 | 0.005 | 2.41E-46 | 0.3022 | -0.0006 | 0.0397 | 0.2639 | 0.9883 |
| rs2296065 | Triglyceride | GALNT2 | A | G | -0.0636 | 0.0069 | 1.54E-20 | 0.161 | 0.0281 | 0.0444 | 0.8084 | 0.5262 |
| rs2678379 | Triglyceride | APOB | G | A | 0.0569 | 0.0059 | 6.43E-22 | 0.2187 | 0.024 | 0.0395 | 0.7338 | 0.544 |
| rs1260326 | Triglyceride | GCKR | C | T | -0.1041 | 0.0051 | 7.70E-92 | 0.5895 | 0.0066 | 0.0366 | 0.6492 | 0.8559 |
| rs59950280 | Triglyceride | HGFAC/DOK7 | A | G | 0.0356 | 0.0055 | 1.10E-10 | 0.3091 | 0.0104 | 0.0411 | 0.2447 | 0.8006 |
| rs3775228 | Triglyceride | AFF1 | T | C | 0.0316 | 0.0051 | 3.81E-10 | 0.3807 | -0.0014 | 0.0357 | 0.3968 | 0.9694 |
| rs28650790 | Triglyceride | C5orf67 | T | C | 0.052 | 0.0068 | 2.34E-14 | 0.8221 | 0.0434 | 0.0507 | 0.1352 | 0.392 |
| rs4704834 | Triglyceride | TIMD4/HAVCR1 | G | A | 0.0339 | 0.0051 | 2.57E-11 | 0.339 | 0.0266 | 0.0373 | 0.6721 | 0.4749 |
| rs35332062 | Triglyceride | MLXIPL | A | G | -0.1162 | 0.0068 | 5.99E-65 | 0.1183 | 0.0353 | 0.0524 | 0.1288 | 0.5004 |
| rs799157 | Triglyceride | MLXIPL | C | T | -0.0951 | 0.0148 | 1.43E-10 | 0.0507 | 0.2022 | 0.1152 | 0.9758 | 0.0792501 |
| rs1364422 | Triglyceride | H4P1/KLF14 | T | C | 0.0371 | 0.0056 | 3.85E-11 | 0.7157 | -0.0197 | 0.0385 | 0.2865 | 0.6093 |
| rs2292369 | Triglyceride | RP11-177H2.1 | G | A | 0.0284 | 0.0051 | 2.12E-08 | 0.5626 | -0.0152 | 0.0395 | 0.3271 | 0.7002 |
| rs112875651 | Triglyceride | LINC00861/TRIB1 | A | G | -0.09 | 0.0051 | 2.27E-69 | 0.3847 | 0.009 | 0.0359 | 0.3872 | 0.8015 |
| rs268 | Triglyceride | LPL | G | A | 0.2531 | 0.0222 | 3.49E-30 | 0.9861 | 0.0585 | 0.1197 | 0.02256 | 0.6252 |
| rs35570672 | Triglyceride | NAT2/PSD3 | C | T | -0.0382 | 0.0061 | 3.10E-10 | 0.7565 | -0.0197 | 0.0403 | 0.7498 | 0.624499 |
| rs74779858 | Triglyceride | LPL | T | C | 0.1638 | 0.0199 | 1.91E-16 | 0.9732 | 0.0074 | 0.1297 | 0.01809 | 0.9547 |
| rs76259755 | Triglyceride | AC100802.3 | C | T | -0.1718 | 0.0073 | 6.40E-124 | 0.1352 | -0.0648 | 0.061 | 0.08969 | 0.2878 |
| rs2068888 | Triglyceride | CYP26A1/NIP7P1 | A | G | -0.0323 | 0.005 | 7.13E-11 | 0.4861 | 0.0096 | 0.035 | 0.4697 | 0.783 |
| rs76836072 | Triglyceride | ZPR1 | T | C | -0.0628 | 0.0098 | 1.77E-10 | 0.9394 | -0.0507 | 0.0756 | 0.05682 | 0.5025 |
| rs174554 | Triglyceride | FADS1/FADS2 | G | A | 0.0375 | 0.0051 | 2.19E-13 | 0.6551 | 0.0014 | 0.0354 | 0.4127 | 0.9688 |
| rs12721078 | Triglyceride | APOA4/APOC3 | A | C | -0.0795 | 0.0139 | 1.18E-08 | 0.0417 | -0.049 | 0.0716 | 0.06257 | 0.4933 |
| rs326222 | Triglyceride | DDB2 | C | T | 0.0326 | 0.0051 | 2.24E-10 | 0.6978 | 0.0249 | 0.0371 | 0.597 | 0.5034 |
| rs3135507 | Triglyceride | APOA5 | T | C | 0.115 | 0.0178 | 1.14E-10 | 0.9652 | 0.0494 | 0.0831 | 0.04558 | 0.552 |
| rs9804646 | Triglyceride | APOA5/APOA4 | T | C | -0.0798 | 0.0086 | 1.43E-20 | 0.9046 | 0.0221 | 0.0523 | 0.1279 | 0.672901 |
| rs61352607 | Triglyceride | INHBC | T | G | -0.0335 | 0.0054 | 6.61E-10 | 0.8052 | -0.0318 | 0.0414 | 0.2331 | 0.4418 |
| rs2262194 | Triglyceride | HCAR3/HCAR2 | G | T | -0.0344 | 0.0059 | 4.92E-09 | 0.7903 | 0.0363 | 0.0445 | 0.1893 | 0.4146 |
| rs7140110 | Triglyceride | GAS6-AS1/GAS6 | C | T | 0.035 | 0.0057 | 7.48E-10 | 0.6849 | 0.0378 | 0.0393 | 0.2801 | 0.337 |
| rs2043085 | Triglyceride | ALDH1A2 | C | T | -0.0322 | 0.0053 | 1.06E-09 | 0.3678 | 0.0244 | 0.0355 | 0.5793 | 0.4911 |
| rs1800588 | Triglyceride | LIPC/ALDH1A2 | T | C | 0.0424 | 0.0064 | 2.44E-11 | 0.7913 | -0.0275 | 0.0403 | 0.2486 | 0.4948 |
| rs1532624 | Triglyceride | CETP | A | C | -0.0274 | 0.0048 | 1.36E-08 | 0.5736 | -0.0255 | 0.0355 | 0.4174 | 0.4717 |
| rs112259268 | Triglyceride | CFAP97D1/MPP3 | A | C | 0.1323 | 0.018 | 1.93E-13 | 0.0348 | 0.0669 | 0.1002 | 0.03157 | 0.5046 |
| rs78357146 | Triglyceride | PRKCA | G | A | -0.0904 | 0.0162 | 2.23E-08 | 0.9592 | 0.2509 | 0.1882 | 0.009402 | 0.1824 |
| rs58542926 | Triglyceride | TM6SF2 | T | C | -0.0879 | 0.0085 | 3.12E-25 | 0.9324 | -0.0384 | 0.0716 | 0.06424 | 0.592199 |
| rs483082 | Triglyceride | APOE/APOC1 | T | G | 0.1095 | 0.0063 | 1.84E-67 | 0.2187 | 0.0206 | 0.0413 | 0.236 | 0.617401 |
| rs116843064 | Triglyceride | ANGPTL4 | A | G | -0.2053 | 0.0145 | 2.00E-45 | 0.0258 | -0.1212 | 0.1085 | 0.02746 | 0.264 |
| rs737338 | Triglyceride | DOCK6 | T | C | -0.0644 | 0.0116 | 3.19E-08 | 0.9573 | 0.0051 | 0.0715 | 0.0635 | 0.9434 |
| rs1057208 | Triglyceride | PCIF1/PLTP | T | C | 0.0447 | 0.0062 | 6.18E-13 | 0.7962 | 0.0238 | 0.0492 | 0.1483 | 0.6282 |
| rs75835816 | ApoA-1 | RPL30P9/LPL | C | G | -0.220950 | 0.03880 | 1.67E-08 | 0.019600 | 0.177 | 0.1227 | 0.02147 | 0.1491 |
| rs1461729 | ApoA-1 | RNU6-526P/RNU6-1151P | G | A | 0.086363 | 0.015193 | 1.77E-08 | 0.861900 | -0.0925 | 0.0504 | 0.8441 | 0.0663101 |
| rs1883025 | ApoA-1 | ABCA1 | T | C | -0.079839 | 0.01332 | 2.86E-09 | 0.194300 | 0.0182 | 0.0443 | 0.1945 | 0.681 |
| rs174594 | ApoA-1 | FADS2 | A | C | 0.071714 | 0.010504 | 1.32E-11 | 0.589200 | -0.007 | 0.0353 | 0.575 | 0.8432 |
| rs113118892 | ApoA-1 | MYO1E | T | C | 0.383240 | 0.056373 | 1.61E-11 | 0.011000 | -0.0154 | 0.1563 | 0.01295 | 0.9214 |
| rs261291 | ApoA-1 | ALDH1A2 | C | T | 0.144314 | 0.010905 | 2.58E-39 | 0.374900 | -0.0338 | 0.0359 | 0.3881 | 0.3466 |
| rs261334 | ApoA-1 | LIPC/ALDH1A2 | C | G | -0.156552 | 0.012564 | 4.81E-35 | 0.769556 | 0.0448 | 0.0411 | 0.7661 | 0.2759 |
| rs247617 | ApoA-1 | HERPUD1/CETP | A | c | 0.197243 | 0.011688 | 7.90E-63 | 0.285998 | -0.0378 | 0.039 | 0.2794 | 0.3316 |
| rs5880 | ApoA-1 | CETP | C | G | -0.186863 | 0.031976 | 6.97E-09 | 0.033156 | 0.0903 | 0.1138 | 0.02585 | 0.4274 |
| rs4939873 | ApoA-1 | LINC02837/LIPG | T | G | 0.134350 | 0.023949 | 2.70E-08 | 0.054342 | -0.0725 | 0.0744 | 0.05736 | 0.3303 |
| rs1168041 | ApoB | DOCK7 | C | T | 0.070994 | 0.011457 | 1.06E-09 | 0.722850 | 0.011 | 0.0396 | 0.7323 | 0.782 |
| rs2495477 | ApoB | PCSK9 | G | A | -0.061897 | 0.01101 | 3.14E-08 | 0.418980 | -0.0272 | 0.0353 | 0.4359 | 0.4414 |
| rs629301 | ApoB | CELSR2 | T | G | 0.090052 | 0.012193 | 3.56E-13 | 0.779917 | 0.0478 | 0.0425 | 0.7854 | 0.2606 |
| rs207179 | ApoB | GOT2P1 | C | T | 0.108802 | 0.017443 | 8.20E-10 | 0.909580 | 0.0326 | 0.0595 | 0.9035 | 0.584 |
| rs11591147 | ApoB | PCSK9 | T | G | -0.437941 | 0.035298 | 2.50E-34 | 0.031100 | -0.0319 | 0.0937 | 0.03608 | 0.733199 |
| rs62123891 | ApoB | AC012361.1 | C | T | -0.099288 | 0.015220 | 1.34E-10 | 0.122222 | -0.0574 | 0.0536 | 0.1203 | 0.2841 |
| rs1260326 | ApoB | GCKR | C | T | -0.066785 | 0.010393 | 2.51E-10 | 0.638553 | 0.0066 | 0.0366 | 0.6492 | 0.8559 |
| rs6756629 | ApoB | ABCG5/ABCG8 | A | G | -0.113253 | 0.018566 | 1.90E-09 | 0.078334 | -0.1036 | 0.0631 | 0.08266 | 0.1007 |
| rs1367117 | ApoB | APOB | A | G | 0.108852 | 0.01119 | 9.99E-22 | 0.287972 | -0.0228 | 0.0388 | 0.281 | 0.5571 |
| rs115849089 | ApoB | RPL30P9/LPL | A | G | -0.099572 | 0.017126 | 1.04E-08 | 0.106662 | -0.0494 | 0.056 | 0.1086 | 0.3772 |
| rs2980875 | ApoB | LINC00861/TRIB1 | G | A | -0.069675 | 0.009993 | 6.68E-12 | 0.483896 | -0.001 | 0.0349 | 0.4669 | 0.9772 |
| rs635634 | ApoB | ABO/Y_RNA | T | C | 0.073971 | 0.012612 | 7.71E-09 | 0.198293 | -0.1 | 0.0435 | 0.2003 | 0.0216701 |
| rs964184 | ApoB | ZPR1 | C | G | -0.165767 | 0.014266 | 2.58E-30 | 0.861008 | 0.0116 | 0.0498 | 0.8541 | 0.8151 |
| rs1081105 | ApoB | APOE/APOC1 | C | A | 0.222868 | 0.03928 | 2.32E-08 | 0.02001 | -0.0194 | 0.1235 | 0.0212 | 0.875 |
| rs7412 | ApoB | APOE | T | C | -0.427553 | 0.025979 | 4.39E-59 | 0.055855 | -0.0292 | 0.079 | 0.05352 | 0.712 |
| rs142130958 | ApoB | LDLR/SMARCA4 | A | G | -0.199648 | 0.016743 | 7.78E-32 | 0.105527 | 0.0315 | 0.0572 | 0.1024 | 0.582599 |
| rs7256200 | ApoB | APOE/APOC1 | T | G | 0.177158 | 0.014426 | 1.15E-33 | 0.15285 | 0.0447 | 0.0474 | 0.1612 | 0.3459 |
| rs1883711 | ApoB | MAFB/LINC01370 | C | G | 0.144092 | 0.02526 | 1.95E-08 | 0.056795 | 0.0723 | 0.0731 | 0.06311 | 0.3225 |
| rs117026595 | Lipoprotein A | LPA | T | A | -0.119870 | 0.012473 | 7.26E-22 | 0.013144 | 0.0589 | 0.1368 | 0.01795 | 0.6669 |
| rs144240142 | Lipoprotein A | MAP3K4 | C | T | -0.273480 | 0.015213 | 3.26E-72 | 0.008439 | -0.1541 | 0.366 | 0.00253 | 0.673701 |
| rs78229704 | Lipoprotein A | AGPAT4/PRKN | G | A | 0.16185 | 0.0095528 | 2.37E-64 | 0.024694 | -0.1407 | 0.1722 | 0.01134 | 0.414 |
| rs143113590 | Lipoprotein A | RP1-155D22.1 | T | C | 0.28369 | 0.0085195 | 1.00E-200 | 0.0262 | 0.0558 | 0.1115 | 0.02552 | 0.6169 |
| rs144177163 | Lipoprotein A | PLG | A | C | -0.232930 | 0.0075723 | 1.00E-200 | 0.0342 | 0.1205 | 0.0981 | 0.03455 | 0.2195 |
| rs112376176 | Lipoprotein A | LPAL2/SLC22A3 | T | C | -0.288070 | 0.0065705 | 1.00E-200 | 0.0438 | -0.2013 | 0.1111 | 0.02543 | 0.0699906 |
| rs10945677 | Lipoprotein A | LPA | T | C | -0.156960 | 0.0033635 | 1.00E-200 | 0.2025 | 0.01 | 0.0403 | 0.2511 | 0.8044 |
| rs118133674 | Lipoprotein A | LPAL2 | A | G | 0.41216 | 0.0093678 | 1.00E-200 | 0.021 | -0.1336 | 0.1535 | 0.01392 | 0.3839 |
| rs2306749 | Lipoprotein A | SYTL3 | G | A | 0.022188 | 0.0029778 | 9.28E-14 | 0.2869 | 0.046 | 0.0417 | 0.2362 | 0.2699 |
| rs75534358 | Lipoprotein A | AGPAT4 | A | G | -0.06737 | 0.0067065 | 9.70E-24 | 0.0422 | 0.1612 | 0.0758 | 0.05734 | 0.0334003 |
| rs12207188 | Lipoprotein A | MAS1 | T | C | 0.27574 | 0.0054966 | 1.00E-200 | 0.0636 | -0.1372 | 0.0806 | 0.05026 | 0.0885809 |
| rs2774229 | Lipoprotein A | SLC22A2 | A | G | -0.125490 | 0.003073 | 1.00E-200 | 0.2602 | -0.0078 | 0.039 | 0.2796 | 0.8422 |
| rs73019670 | Lipoprotein A | IGF2R | A | G | -0.112300 | 0.005067 | 9.93E-109 | 0.0769 | 0.0041 | 0.0686 | 0.07061 | 0.9519 |
| rs77613626 | Lipoprotein A | LPAL2 | G | C | -0.424460 | 0.013008 | 1.00E-200 | 0.0116 | -0.0155 | 0.1563 | 0.01347 | 0.9212 |
| rs77772165 | Lipoprotein A | PARK2 | C | T | -0.115480 | 0.014522 | 1.84E-15 | 0.0095 | 1.586 | 0.6923 | 0.0008979 | 0.02197 |
| rs4646272 | Lipoprotein A | SLC22A1 | G | T | 0.32731 | 0.0060116 | 1.00E-200 | 0.0531 | -0.1442 | 0.0731 | 0.06241 | 0.0483704 |
| rs41272114 | Lipoprotein A | LPA | T | C | -0.367770 | 0.0082137 | 1.00E-200 | 0.0283 | 0.0739 | 0.0821 | 0.04686 | 0.3682 |
| rs143048975 | Lipoprotein A | PLG | A | G | 0.40746 | 0.007387 | 1.00E-200 | 0.0414 | -0.03 | 0.0884 | 0.04218 | 0.734401 |
| rs76252099 | Lipoprotein A | FNDC1 | A | G | 0.049046 | 0.0048785 | 8.94E-24 | 0.0835 | 0.069 | 0.0737 | 0.05926 | 0.3491 |
| rs73017498 | Lipoprotein A | AGPAT4 | C | G | -0.087093 | 0.010019 | 3.57E-18 | 0.0189 | 0.1976 | 0.1146 | 0.02431 | 0.0845201 |
| rs56203233 | Lipoprotein A | RP1-155D22.1 | A | G | 0.10239 | 0.0065952 | 2.49E-54 | 0.048 | 0.0637 | 0.0853 | 0.04782 | 0.4553 |
| rs73027919 | Lipoprotein A | LINC02529/FNDC1 | T | C | -0.066080 | 0.0070186 | 4.77E-21 | 0.0385 | 0.0446 | 0.1167 | 0.02474 | 0.702399 |
| rs3912161 | Lipoprotein A | SLC22A2 | C | T | -0.100940 | 0.0058574 | 1.63E-66 | 0.0594 | -0.0008 | 0.0621 | 0.08842 | 0.9894 |
| rs76018427 | Lipoprotein A | SLC22A3 | C | T | 0.10577 | 0.009745 | 1.95E-27 | 0.0197 | 0.0381 | 0.0779 | 0.05268 | 0.6248 |
| rs139141144 | Lipoprotein A | SNX9 | A | G | 0.056257 | 0.010088 | 2.45E-08 | 0.0203 | 0.2216 | 0.208 | 0.007352 | 0.2866 |
| rs150960004 | Lipoprotein A | SLC22A3 | T | C | -0.196180 | 0.0093965 | 1.02E-96 | 0.0215 | -0.0077 | 0.1031 | 0.03014 | 0.9404 |
| rs34498812 | Lipoprotein A | LPA | A | G | -0.253570 | 0.0085993 | 8.32E-19 | 0.0253 | 0.05 | 0.0952 | 0.03737 | 0.5995 |
| rs147082524 | Lipoprotein A | SLC22A1 | T | C | -0.164920 | 189060 | 2.73E-18 | 0.0056 | -0.3508 | 0.3377 | 0.002822 | 0.2988 |
| rs139947401 | Lipoprotein A | MAP3K4 | T | C | -0.211250 | 0.011833 | 3.08E-07 | 0.0135 | 0.6651 | 0.2454 | 0.005723 | 0.00672806 |
| rs4282384 | Lipoprotein A | PARK2 | T | C | 0.091158 | 0.0067969 | 5.32E-41 | 0.0477 | -0.0117 | 0.0629 | 0.08815 | 0.853 |
| rs145221006 | Lipoprotein A | SLC22A2 | A | G | -0.249950 | 0.0051216 | 1.00E-200 | 0.0811 | 0.1282 | 0.0651 | 0.08027 | 0.0487899 |
| rs148623260 | Lipoprotein A | RP1-155D22.1 | A | G | 0.18413 | 0.011505 | 1.27E-57 | 0.0164 | -0.1083 | 0.1484 | 0.01478 | 0.4655 |
| rs9355755 | Lipoprotein A | RP1-155D22.1 | C | T | 0.061065 | 0.0066833 | 6.46E-20 | 0.0432 | 0.0871 | 0.0824 | 0.04712 | 0.2908 |
| rs117901488 | Lipoprotein A | SLC22A2 | T | C | 0.23782 | 0.0062448 | 1.00E-200 | 0.0489 | -0.0286 | 0.1026 | 0.02927 | 0.7802 |
| rs74956805 | Lipoprotein A | RP1-155D22.1 | A | G | -0.072772 | 0.0050853 | 1.96E-46 | 0.0785 | -0.0115 | 0.0806 | 0.05026 | 0.8861 |
| rs146752622 | Lipoprotein A | AGPAT4 | T | G | -0.098359 | 0.0098053 | 1.12E-23 | 0.022 | 0.0446 | 0.0779 | 0.01468 | 0.3395 |
| rs62637702 | Lipoprotein A | PARK2 | C | T | 0.16804 | 0.011486 | 1.89E-48 | 0.0165 | 0.1446 | 0.1514 | 0.0391 | 0.2007 |
| rs73023871 | Lipoprotein A | RP3-428L16.2 | G | A | -0.140740 | 0.014182 | 3.33E-23 | 0.0093 | -0.0033 | 0.1575 | 0.01294 | 0.9835 |
| rs12446515 | Lipoprotein A | HERPUD1/CETP | T | C | -0.024071 | 0.0028945 | 9.14E-17 | 0.3236 | -0.0371 | 0.0391 | 0.2797 | 0.3423 |
| rs8178824 | Lipoprotein A | APOH | T | C | 0.05347 | 0.007964 | 1.90E-11 | 0.0298 | 0.2398 | 0.1874 | 0.00946 | 0.2007 |
